# Supplementary figures and images for: A UAV Thermal Imaging Format Conversion System and Its Application in Mosaic Surface Microthermal Environment Analysis
Source: Sensors (Basel). 2024 Sep 27;24(19):6267. doi: 10.3390/s24196267 (PMC11479196; doi:10.3390/s24196267)

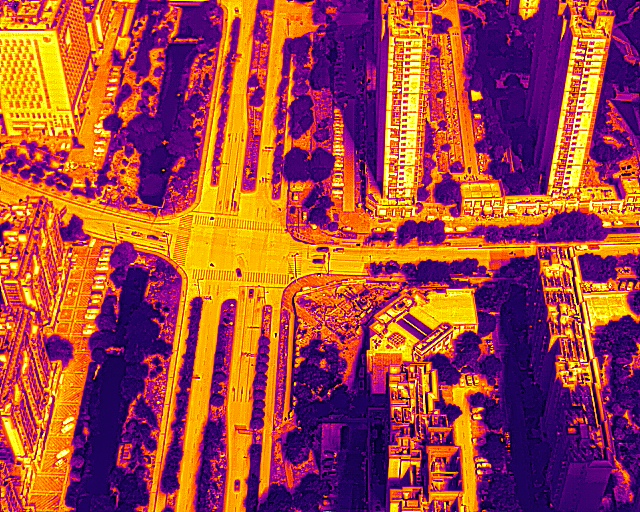

Supplement: Supplementary file 1 [file sensors-24-06267-s001.zip › sensors-3194209-supplementary/ThermoSwitcher/DJI_20240321141556_0001_T.JPG]

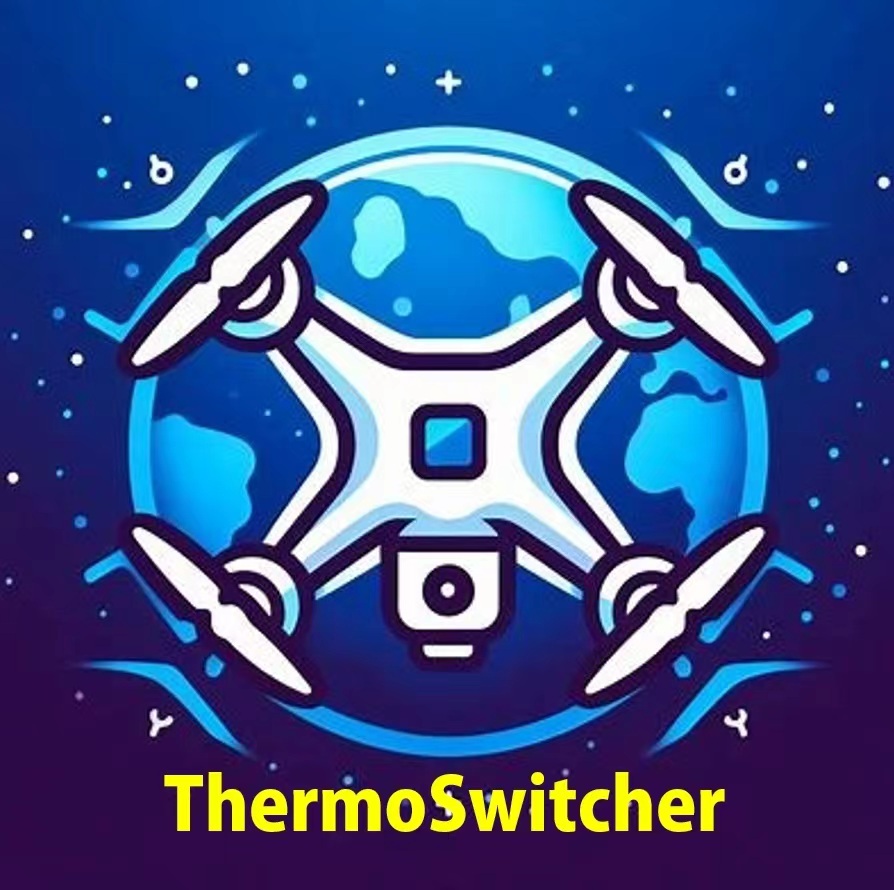

Supplement: Supplementary file 1 [file sensors-24-06267-s001.zip › sensors-3194209-supplementary/ThermoSwitcher/icon.jpg]

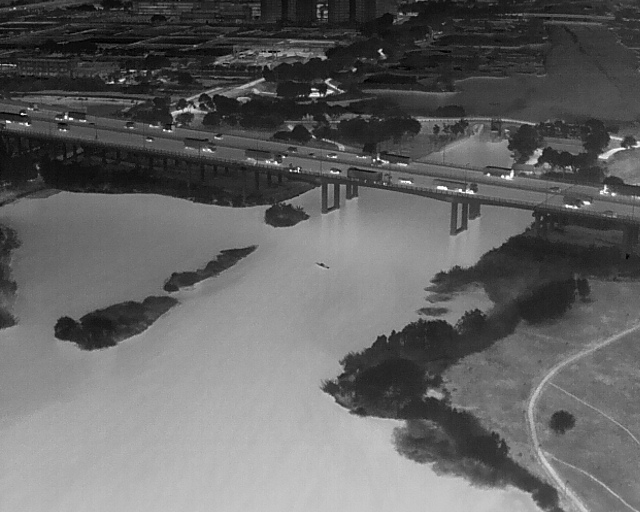

Supplement: Supplementary file 1 [file sensors-24-06267-s001.zip › sensors-3194209-supplementary/ThermoSwitcher/sample_dataset/H20N/DJI_0001_R.JPG]

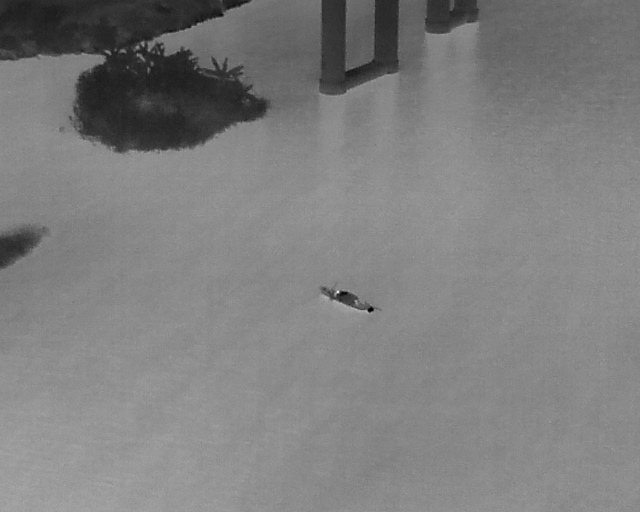

Supplement: Supplementary file 1 [file sensors-24-06267-s001.zip › sensors-3194209-supplementary/ThermoSwitcher/sample_dataset/H20N/DJI_0002_R.JPG]

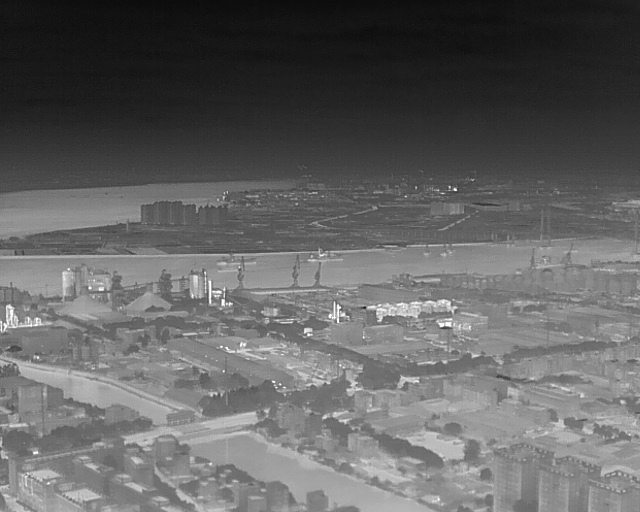

Supplement: Supplementary file 1 [file sensors-24-06267-s001.zip › sensors-3194209-supplementary/ThermoSwitcher/sample_dataset/H20N/DJI_0003_R.JPG]

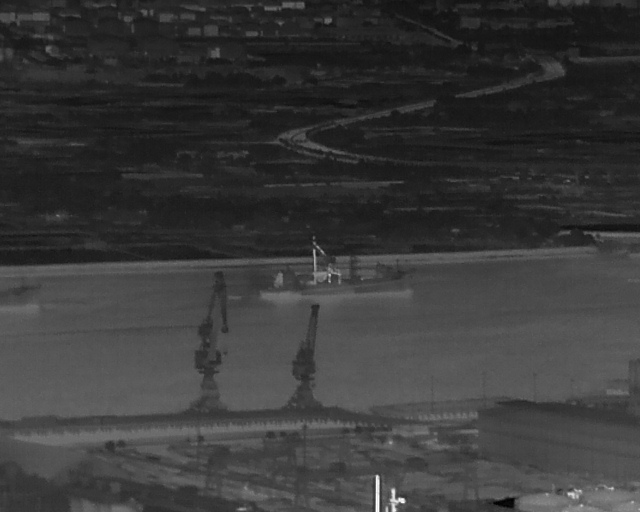

Supplement: Supplementary file 1 [file sensors-24-06267-s001.zip › sensors-3194209-supplementary/ThermoSwitcher/sample_dataset/H20N/DJI_0004_R.JPG]

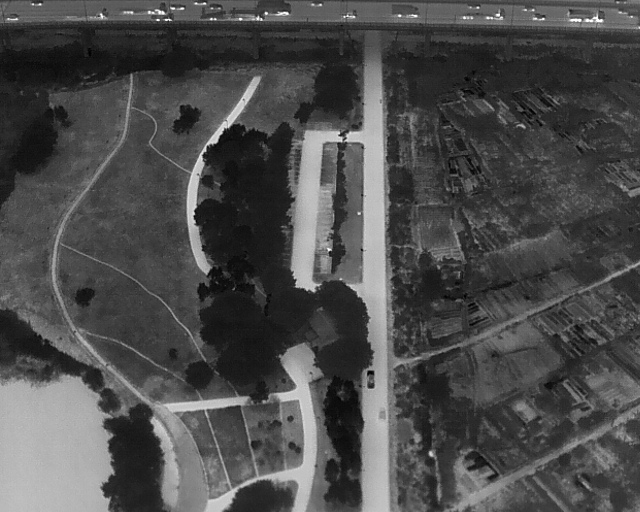

Supplement: Supplementary file 1 [file sensors-24-06267-s001.zip › sensors-3194209-supplementary/ThermoSwitcher/sample_dataset/H20N/DJI_0005_R.JPG]

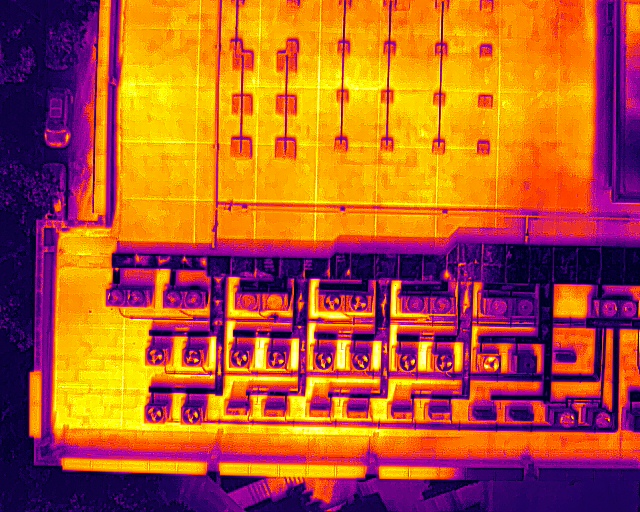

Supplement: Supplementary file 1 [file sensors-24-06267-s001.zip › sensors-3194209-supplementary/ThermoSwitcher/sample_dataset/H20T/DJI_0001_R.JPG]

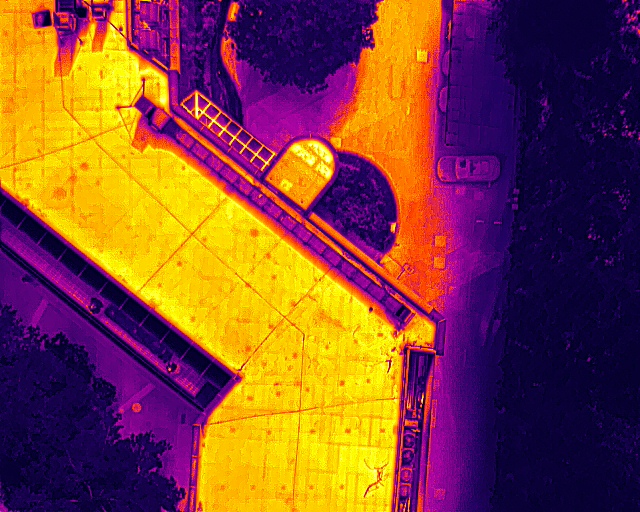

Supplement: Supplementary file 1 [file sensors-24-06267-s001.zip › sensors-3194209-supplementary/ThermoSwitcher/sample_dataset/H20T/DJI_0002_R.JPG]

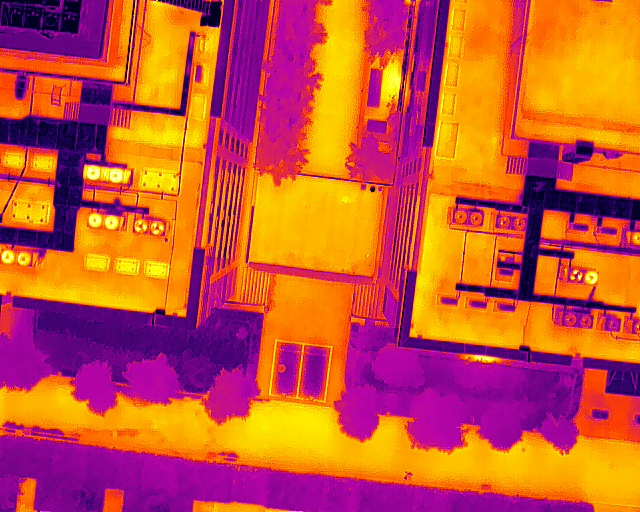

Supplement: Supplementary file 1 [file sensors-24-06267-s001.zip › sensors-3194209-supplementary/ThermoSwitcher/sample_dataset/H20T/DJI_0003_R.JPG]

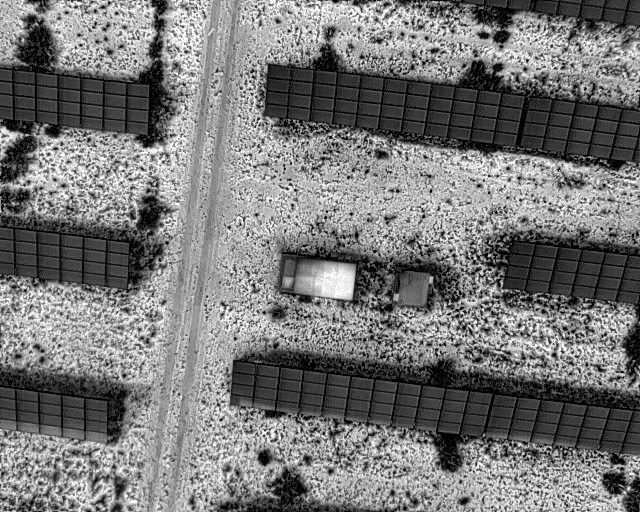

Supplement: Supplementary file 1 [file sensors-24-06267-s001.zip › sensors-3194209-supplementary/ThermoSwitcher/sample_dataset/H20T/DJI_0004_R.JPG]

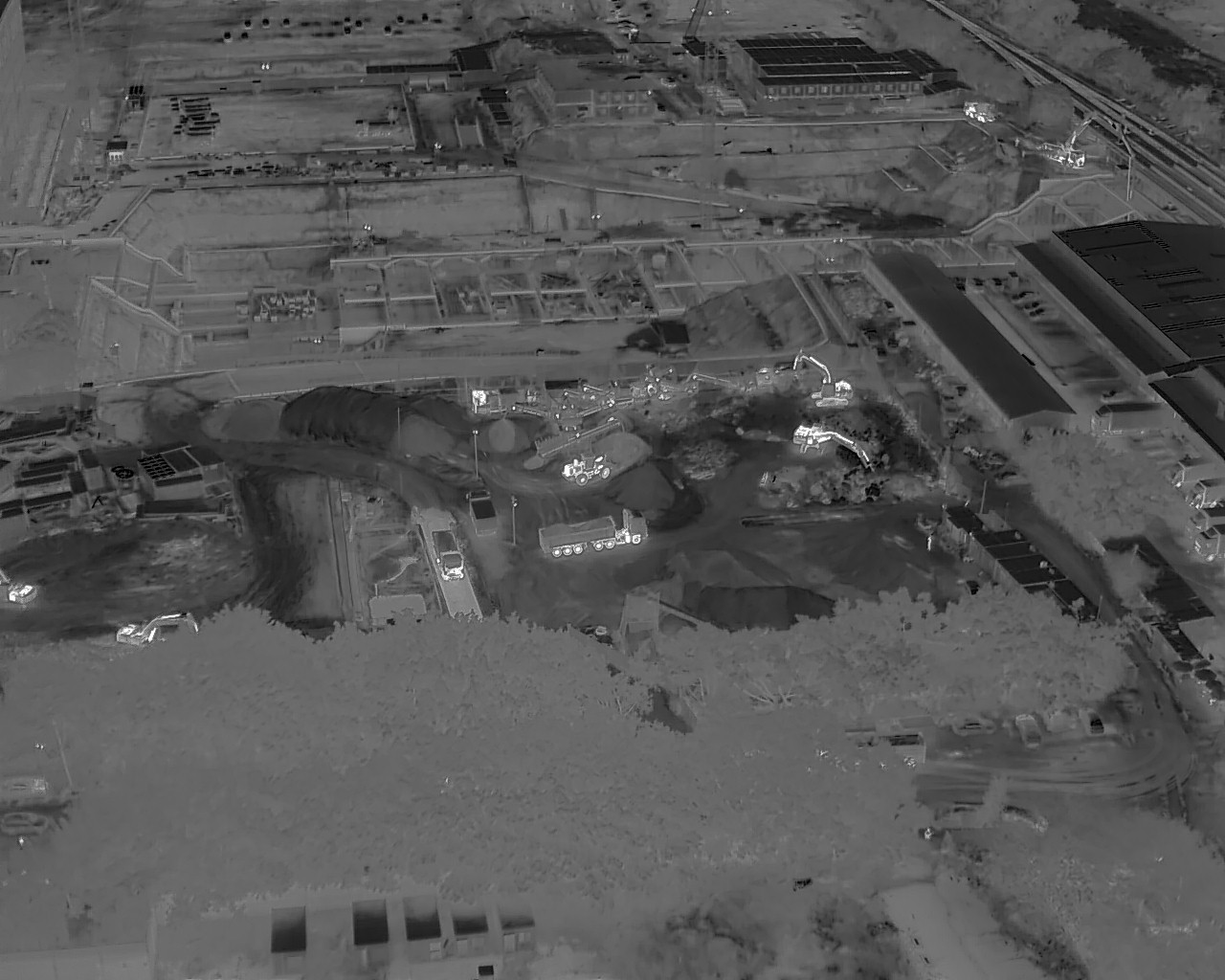

Supplement: Supplementary file 1 [file sensors-24-06267-s001.zip › sensors-3194209-supplementary/ThermoSwitcher/sample_dataset/H30T/DJI_0001_R.JPG]

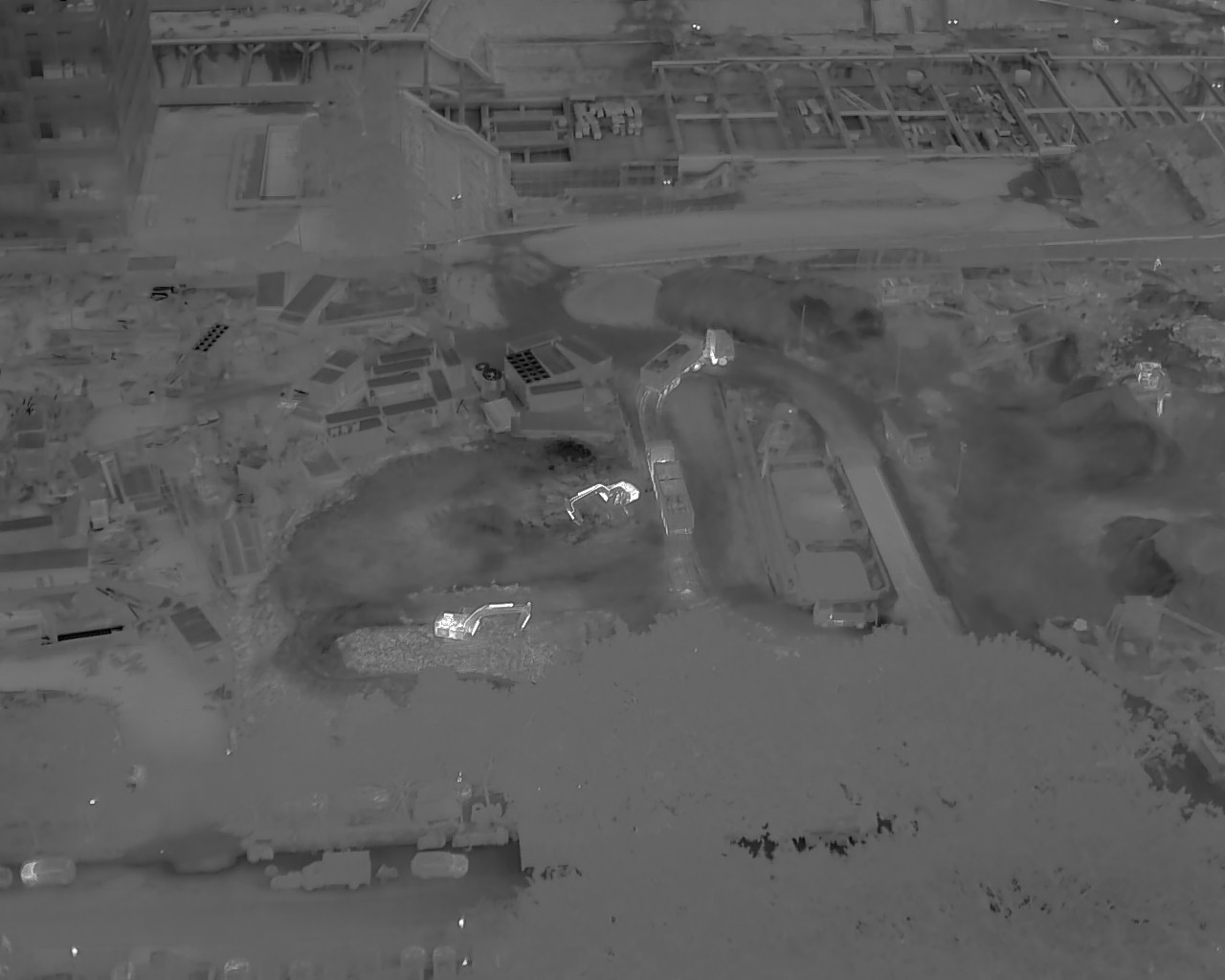

Supplement: Supplementary file 1 [file sensors-24-06267-s001.zip › sensors-3194209-supplementary/ThermoSwitcher/sample_dataset/H30T/DJI_0002_R.JPG]

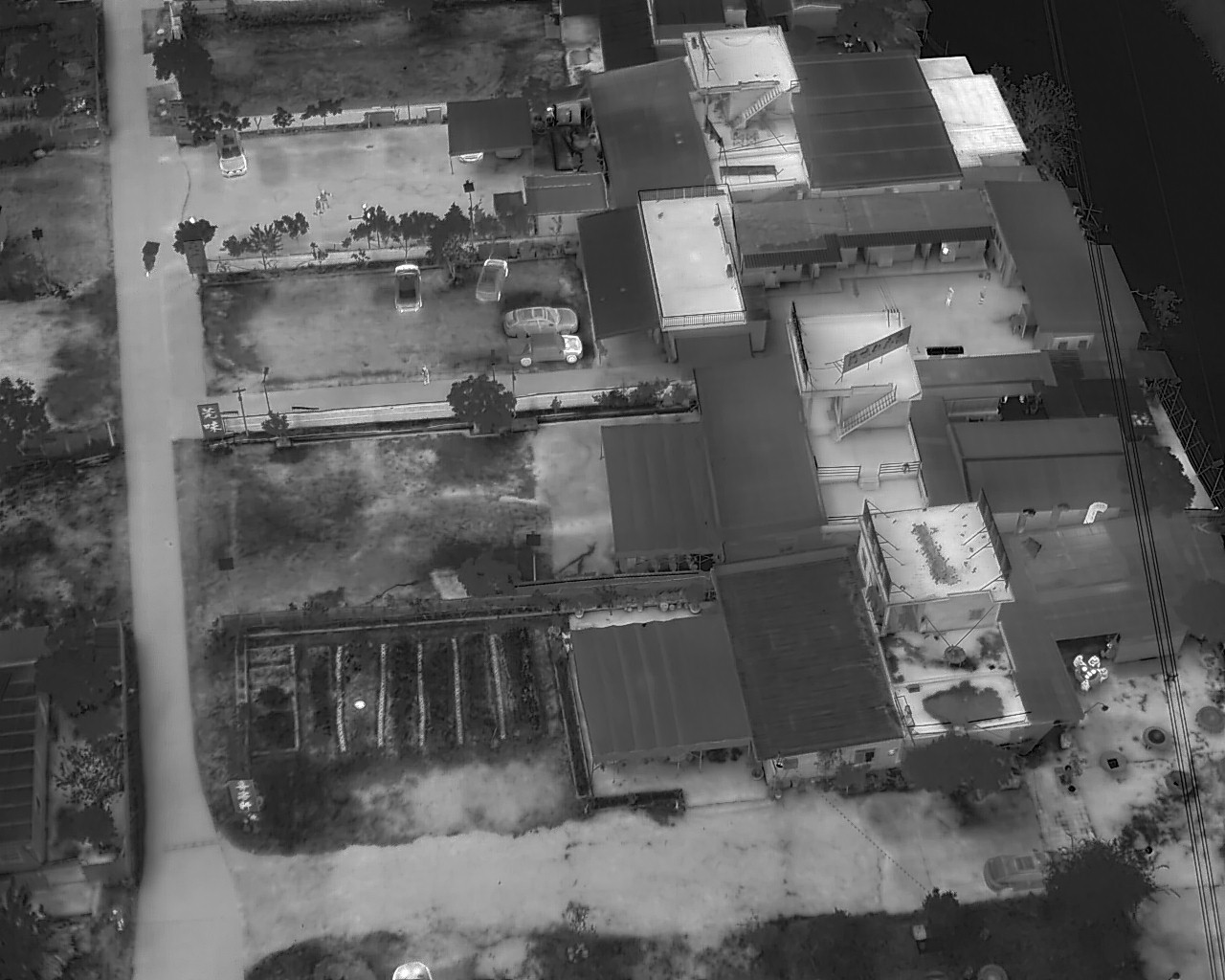

Supplement: Supplementary file 1 [file sensors-24-06267-s001.zip › sensors-3194209-supplementary/ThermoSwitcher/sample_dataset/H30T/DJI_0003_R.JPG]

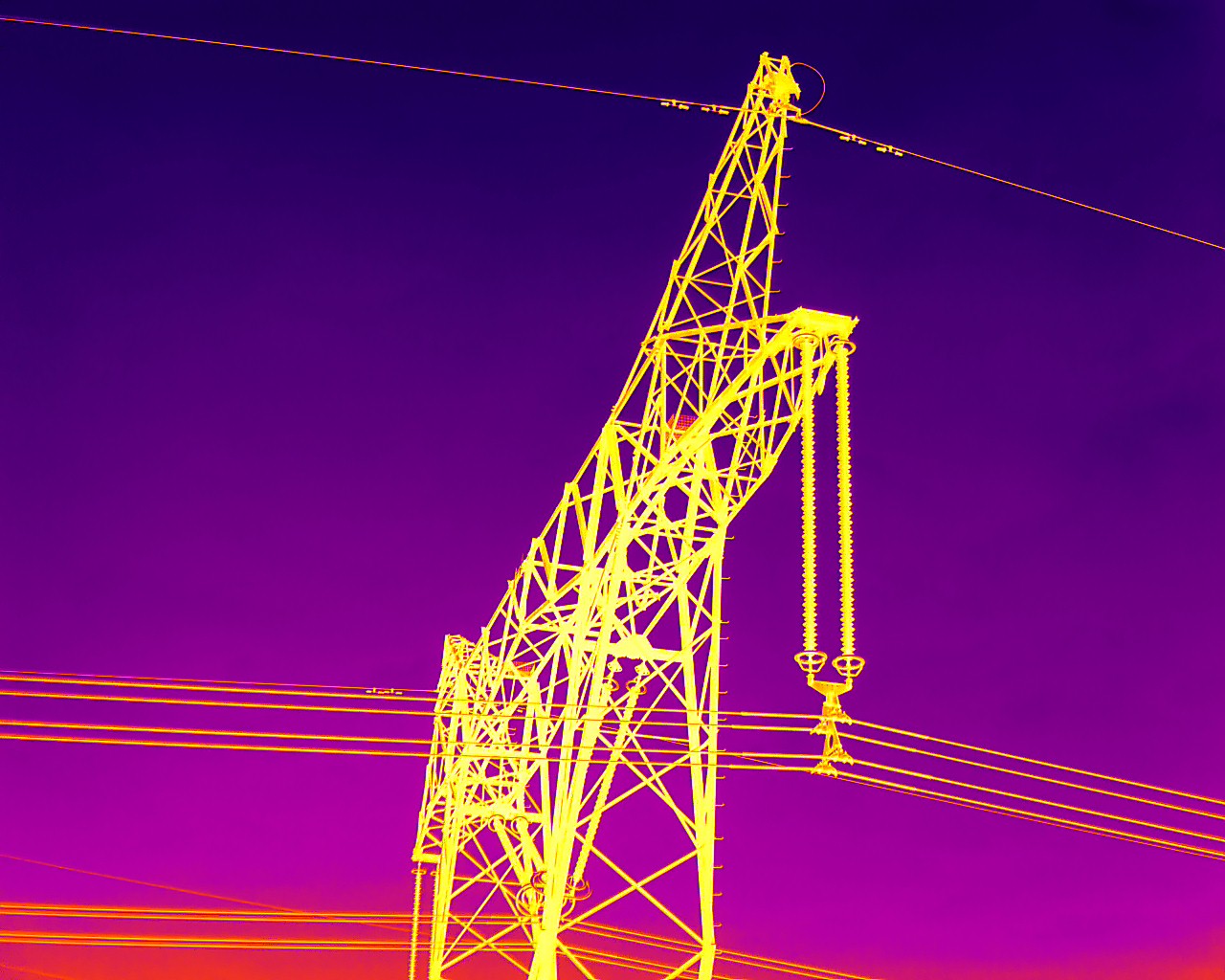

Supplement: Supplementary file 1 [file sensors-24-06267-s001.zip › sensors-3194209-supplementary/ThermoSwitcher/sample_dataset/H30T/DJI_0004_R.JPG]

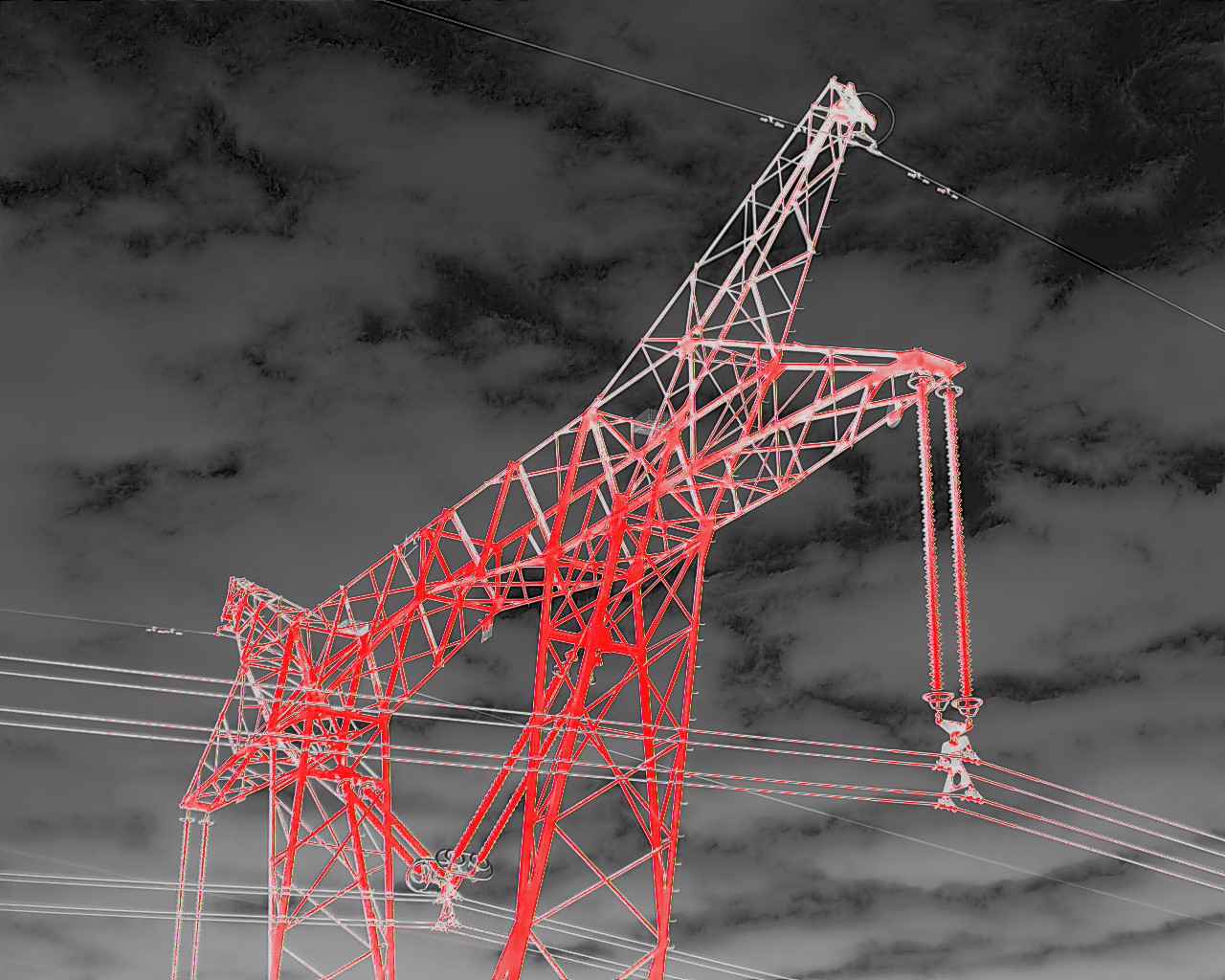

Supplement: Supplementary file 1 [file sensors-24-06267-s001.zip › sensors-3194209-supplementary/ThermoSwitcher/sample_dataset/H30T/DJI_0005_R.JPG]

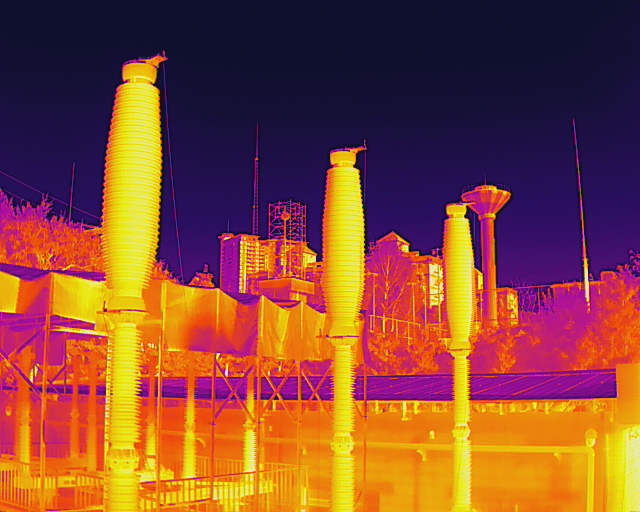

Supplement: Supplementary file 1 [file sensors-24-06267-s001.zip › sensors-3194209-supplementary/ThermoSwitcher/sample_dataset/M2EA/DJI_0001_R.JPG]

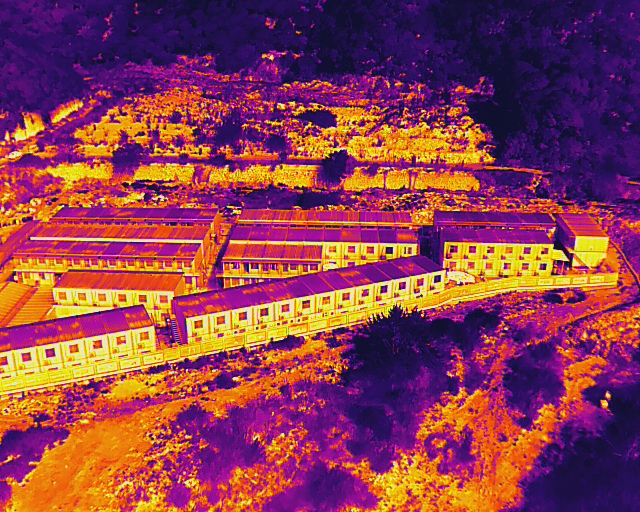

Supplement: Supplementary file 1 [file sensors-24-06267-s001.zip › sensors-3194209-supplementary/ThermoSwitcher/sample_dataset/M2EA/DJI_0002_R.JPG]

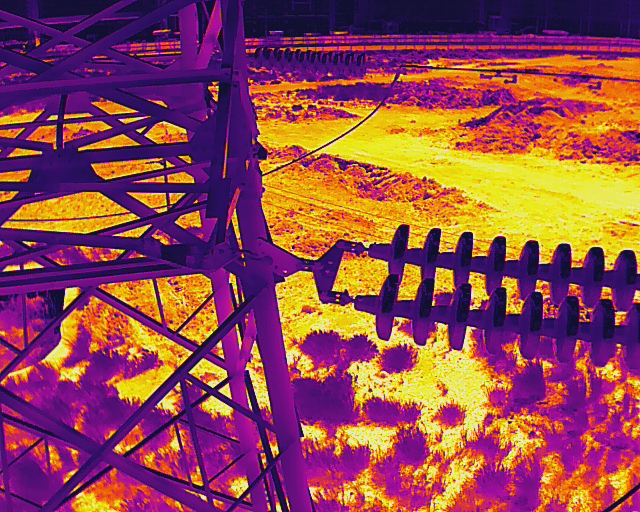

Supplement: Supplementary file 1 [file sensors-24-06267-s001.zip › sensors-3194209-supplementary/ThermoSwitcher/sample_dataset/M2EA/DJI_0003_R.JPG]

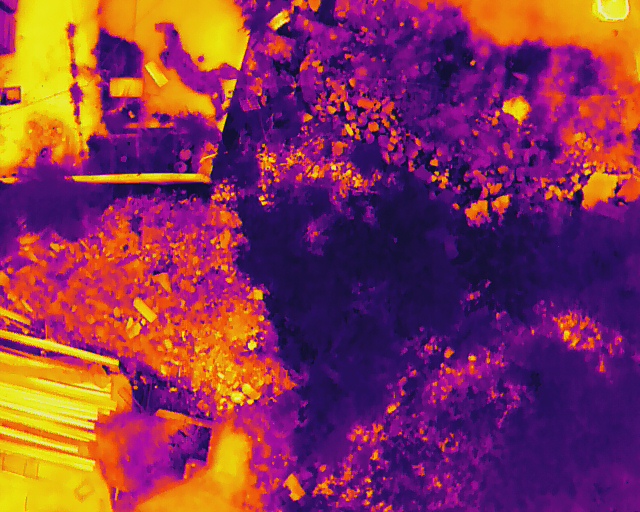

Supplement: Supplementary file 1 [file sensors-24-06267-s001.zip › sensors-3194209-supplementary/ThermoSwitcher/sample_dataset/M2EA/DJI_0004_R.JPG]

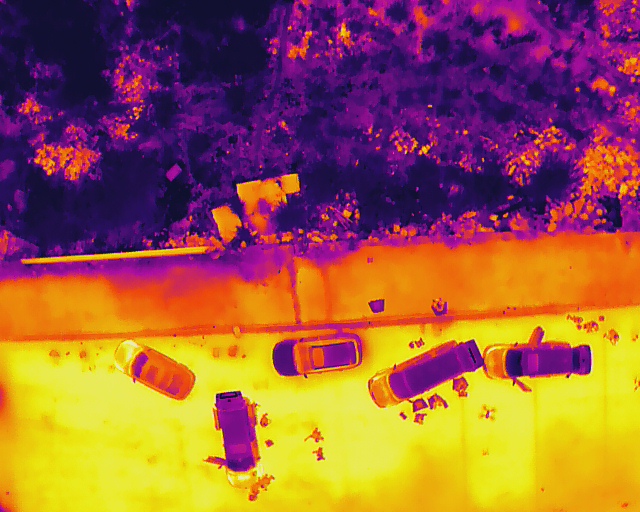

Supplement: Supplementary file 1 [file sensors-24-06267-s001.zip › sensors-3194209-supplementary/ThermoSwitcher/sample_dataset/M2EA/DJI_0005_R.JPG]

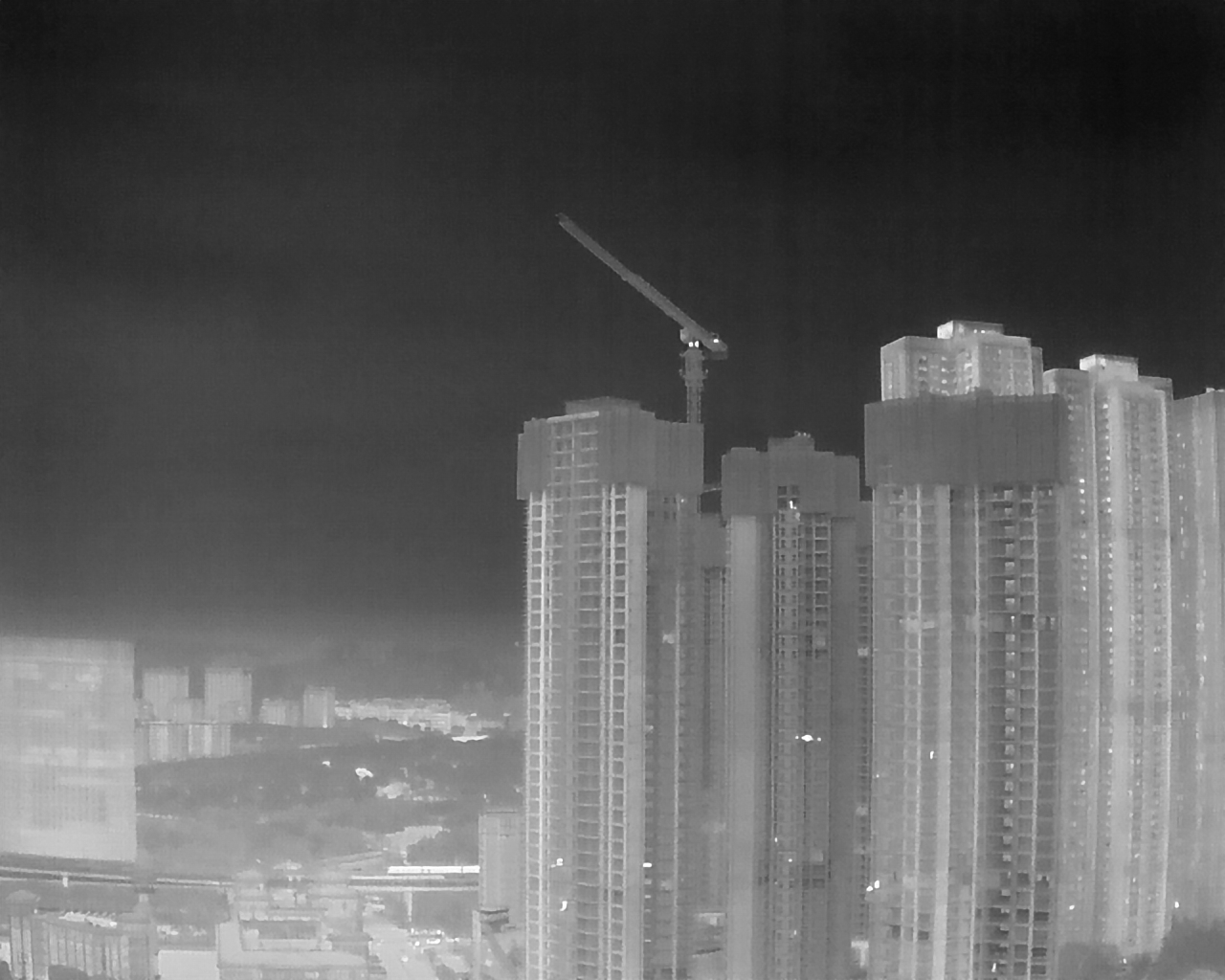

Supplement: Supplementary file 1 [file sensors-24-06267-s001.zip › sensors-3194209-supplementary/ThermoSwitcher/sample_dataset/M30T/DJI_0001_R.JPG]

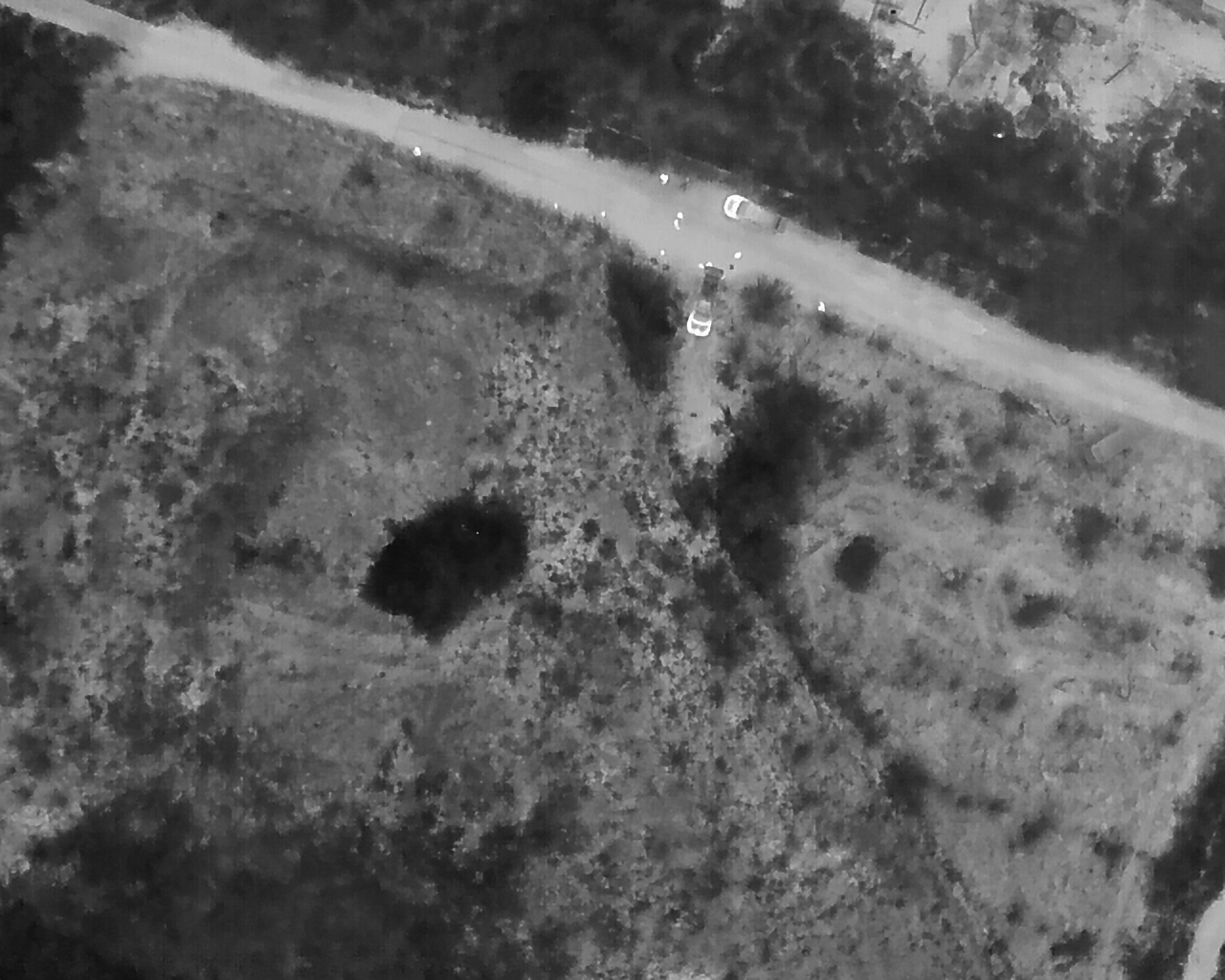

Supplement: Supplementary file 1 [file sensors-24-06267-s001.zip › sensors-3194209-supplementary/ThermoSwitcher/sample_dataset/M30T/DJI_0002_R.JPG]

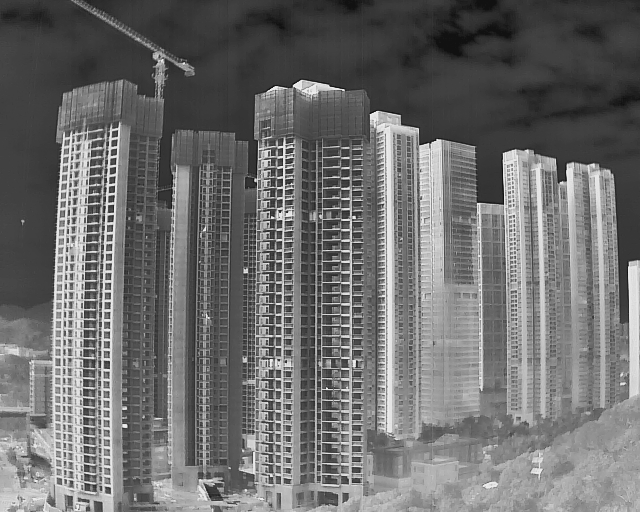

Supplement: Supplementary file 1 [file sensors-24-06267-s001.zip › sensors-3194209-supplementary/ThermoSwitcher/sample_dataset/M30T/DJI_0003_R.JPG]

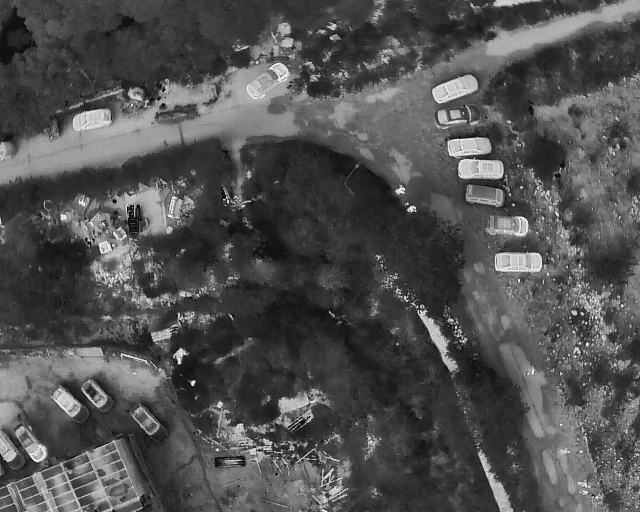

Supplement: Supplementary file 1 [file sensors-24-06267-s001.zip › sensors-3194209-supplementary/ThermoSwitcher/sample_dataset/M30T/DJI_0004_R.JPG]

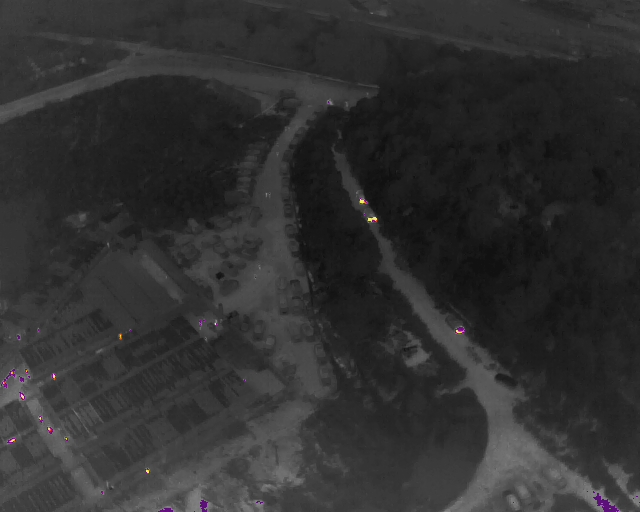

Supplement: Supplementary file 1 [file sensors-24-06267-s001.zip › sensors-3194209-supplementary/ThermoSwitcher/sample_dataset/M30T/DJI_0005_R.JPG]

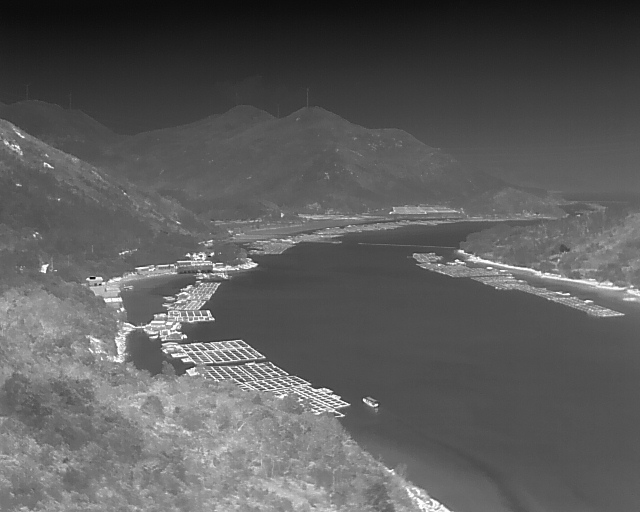

Supplement: Supplementary file 1 [file sensors-24-06267-s001.zip › sensors-3194209-supplementary/ThermoSwitcher/sample_dataset/M3T/DJI_0001_R.JPG]

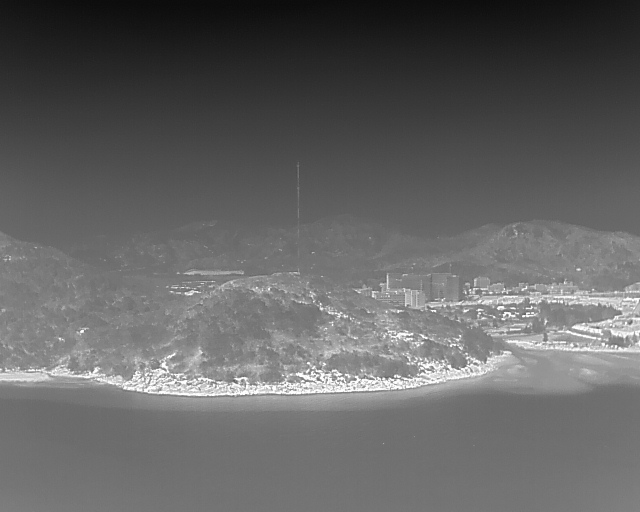

Supplement: Supplementary file 1 [file sensors-24-06267-s001.zip › sensors-3194209-supplementary/ThermoSwitcher/sample_dataset/M3T/DJI_0002_R.JPG]

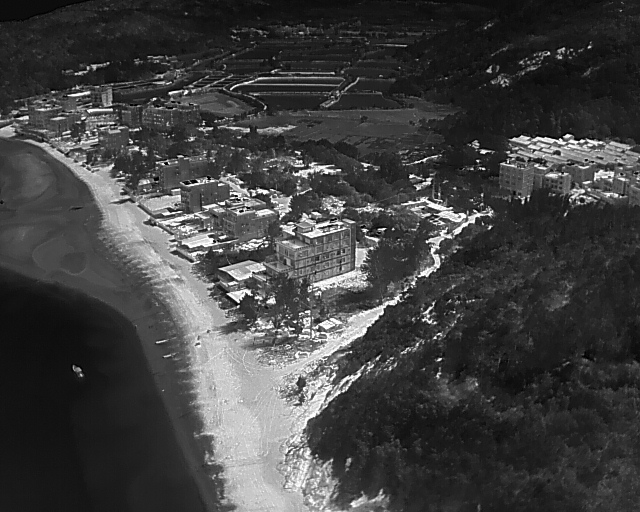

Supplement: Supplementary file 1 [file sensors-24-06267-s001.zip › sensors-3194209-supplementary/ThermoSwitcher/sample_dataset/M3T/DJI_0003_R.JPG]

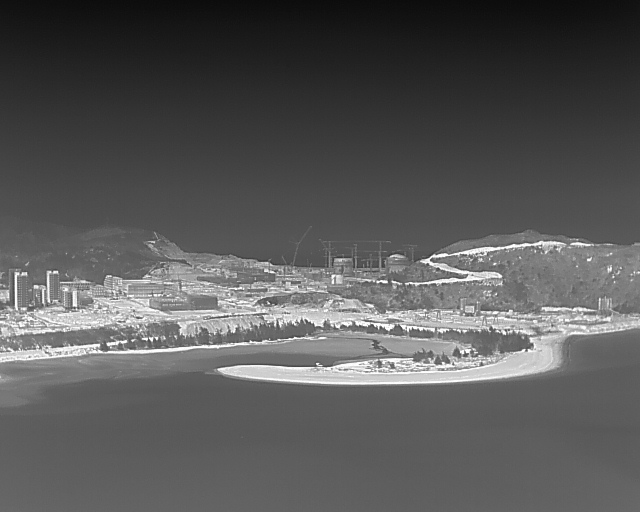

Supplement: Supplementary file 1 [file sensors-24-06267-s001.zip › sensors-3194209-supplementary/ThermoSwitcher/sample_dataset/M3T/DJI_0004_R.JPG]

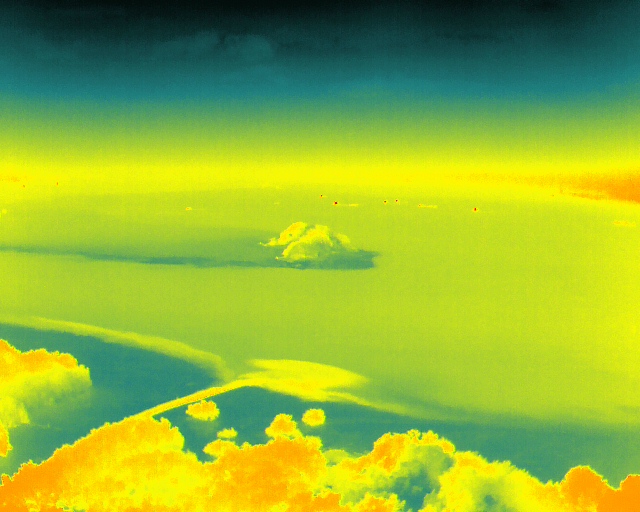

Supplement: Supplementary file 1 [file sensors-24-06267-s001.zip › sensors-3194209-supplementary/ThermoSwitcher/sample_dataset/M3T/DJI_0005_R.JPG]

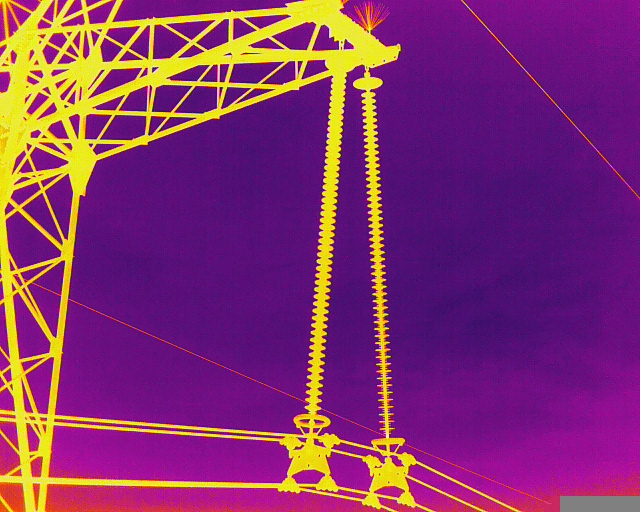

Supplement: Supplementary file 1 [file sensors-24-06267-s001.zip › sensors-3194209-supplementary/ThermoSwitcher/sample_dataset/XTS/DJI_0001_R.jpg]

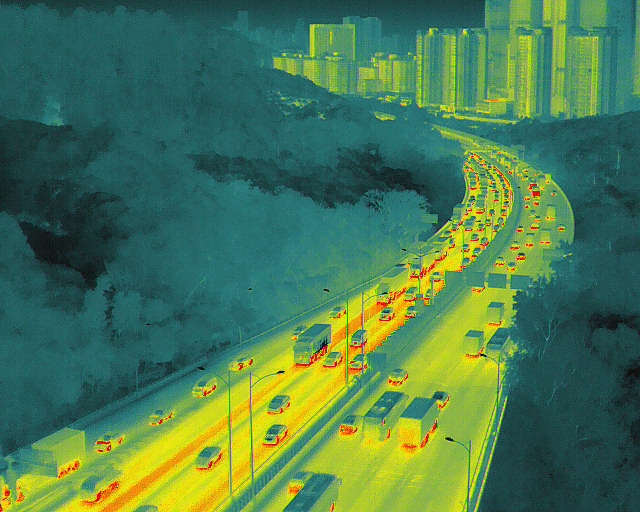

Supplement: Supplementary file 1 [file sensors-24-06267-s001.zip › sensors-3194209-supplementary/ThermoSwitcher/sample_dataset/XTS/DJI_0002_R.jpg]

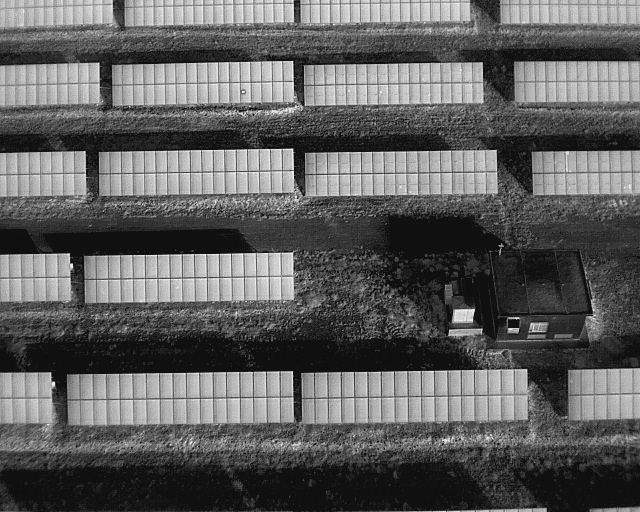

Supplement: Supplementary file 1 [file sensors-24-06267-s001.zip › sensors-3194209-supplementary/ThermoSwitcher/sample_dataset/XTS/DJI_0003_R.JPG]

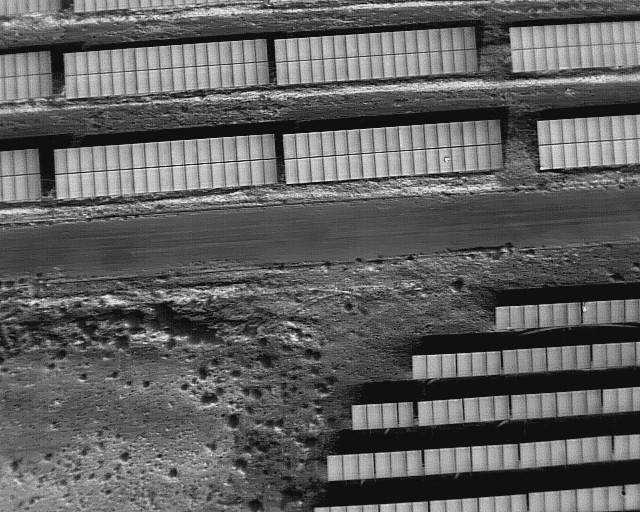

Supplement: Supplementary file 1 [file sensors-24-06267-s001.zip › sensors-3194209-supplementary/ThermoSwitcher/sample_dataset/XTS/DJI_0004_R.JPG]

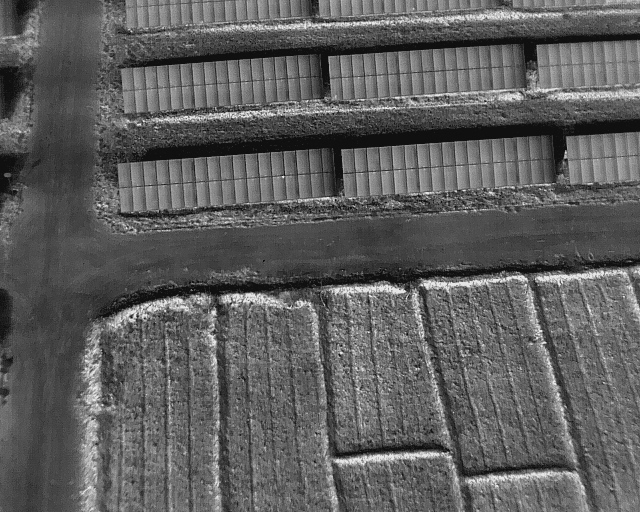

Supplement: Supplementary file 1 [file sensors-24-06267-s001.zip › sensors-3194209-supplementary/ThermoSwitcher/sample_dataset/XTS/DJI_0005_R.JPG]
